# Vasodilator-Stimulated Phosphoprotein Activity Is Required for *Coxiella burnetii* Growth in Human Macrophages

**DOI:** 10.1371/journal.ppat.1005915

**Published:** 2016-10-06

**Authors:** Punsiri M. Colonne, Caylin G. Winchell, Joseph G. Graham, Frances I. Onyilagha, Laura J. MacDonald, Heike R. Doeppler, Peter Storz, Richard C. Kurten, Paul A. Beare, Robert A. Heinzen, Daniel E. Voth

**Affiliations:** 1 Department of Microbiology and Immunology, University of Arkansas for Medical Sciences, Little Rock, Arkansas, United States of America; 2 Department of Cancer Biology, Mayo Clinic, Jacksonville, Florida, United States of America; 3 Department of Physiology and Biophysics, University of Arkansas for Medical Sciences, Little Rock, Arkansas, United States of America; 4 Arkansas Children’s Hospital Research Institute, Little Rock, Arkansas, United States of America; 5 Coxiella Pathogenesis Section, Laboratory of Bacteriology, Rocky Mountain Laboratories, National Institute of Allergy and Infectious Diseases, National Institutes of Health, Hamilton, Montana, United States of America; Purdue University, UNITED STATES

## Abstract

*Coxiella burnetii* is an intracellular bacterial pathogen that causes human Q fever, an acute flu-like illness that can progress to chronic endocarditis and liver and bone infections. Humans are typically infected by aerosol-mediated transmission, and *C*. *burnetii* initially targets alveolar macrophages wherein the pathogen replicates in a phagolysosome-like niche known as the parasitophorous vacuole (PV). *C*. *burnetii* manipulates host cAMP-dependent protein kinase (PKA) signaling to promote PV formation, cell survival, and bacterial replication. In this study, we identified the actin regulatory protein vasodilator-stimulated phosphoprotein (VASP) as a PKA substrate that is increasingly phosphorylated at S157 and S239 during *C*. *burnetii* infection. Avirulent and virulent *C*. *burnetii* triggered increased levels of phosphorylated VASP in macrophage-like THP-1 cells and primary human alveolar macrophages, and this event required the Cα subunit of PKA. VASP phosphorylation also required bacterial protein synthesis and secretion of effector proteins via a type IV secretion system, indicating the pathogen actively triggers prolonged VASP phosphorylation. Optimal PV formation and intracellular bacterial replication required VASP activity, as siRNA-mediated depletion of VASP reduced PV size and bacterial growth. Interestingly, ectopic expression of a phospho-mimetic VASP (S239E) mutant protein prevented optimal PV formation, whereas VASP (S157E) mutant expression had no effect. VASP (S239E) expression also prevented trafficking of bead-containing phagosomes to the PV, indicating proper VASP activity is critical for heterotypic fusion events that control PV expansion in macrophages. Finally, expression of dominant negative VASP (S157A) in *C*. *burnetii*-infected cells impaired PV formation, confirming importance of the protein for proper infection. This study provides the first evidence of VASP manipulation by an intravacuolar bacterial pathogen via activation of PKA in human macrophages.

## Author Summary

Q fever, caused by the intracellular bacterial pathogen *Coxiella burnetii*, is an aerosol-transmitted infection that can develop into life-threatening chronic infections such as endocarditis. The pathogen preferentially grows within alveolar macrophages in a phagolysosome-like compartment termed the parasitophorous vacuole (PV). *C*. *burnetii* actively manipulates host cAMP-dependent protein kinase (PKA) signaling to promote PV formation and cell survival. Identification of bacterial effector proteins that manipulate PKA and downstream target proteins is critical to fully understand pathogen-mediated signaling circuits and develop new therapeutic strategies. Here, we found that PKA controls vasodilator-stimulated phosphoprotein (VASP) activity to promote PV formation and bacterial replication. VASP regulates actin-based motility used by a subset of intracellular bacteria for propulsion through the host cell cytosol and into bystander cells. However, *C*. *burnetii* does not use actin-based motility and replicates throughout its life cycle within a membrane bound vacuole. Thus, this study provides the first evidence of VASP manipulation by an intravacuolar bacterial pathogen. Characterization of VASP function in PV formation and identification of additional PKA substrates that promote infection will provide new insight into host-pathogen interactions during Q fever.

## Introduction

*Coxiella burnetii* is an intracellular bacterial pathogen that causes the zoonosis human Q fever. *C*. *burnetii* infects domestic mammals and livestock, which serve as the primary reservoir for the pathogen in nature. *C*. *burnetii* is shed from infected animals in body fluids, particularly during parturition, resulting in human infection by inhalation of contaminated aerosols [1]. Q fever often manifests as a flu-like acute disease with atypical pneumonia, and most individuals recover without medical intervention. However, less than 5% of infected individuals develop chronic disease that largely manifests as endocarditis, and to a lesser extent as bone infection, vascular complications, and granulomatous hepatitis [2]. The fatality rate of patients with Q fever endocarditis approaches 60% if left untreated [1]. Chronic Q fever diagnosis is extremely difficult and treatment requires a prolonged course of antibiotic therapy that is not completely effective. Considering the global distribution of *C*. *burnetii*, and recent outbreaks in rural parts of the world, Q fever is now considered an emerging infectious disease [3,4].

In aerosol-acquired human infections, *C*. *burnetii* preferentially targets macrophages that reside in alveolar spaces. Virulent bacteria enter macrophages by α_V_β_3_ integrin receptor-dependent phagocytosis [5]. Following invasion, organisms reside in tight-fitting phagosomes that decorate with the autophagosome marker LC3 and early endosomal Rab5 [6]. Maturation of nascent phagosomes into unique, replication-permissive parasitophorous vacuoles (PV) is achieved by continual heterotypic fusion with autophagosomes, endosomes, and lysosomes [7–9]. Although lysosome fusion creates an acidic, hydrolytic environment, *C*. *burnetii* has adapted to resist degradation, and low pH activates bacterial metabolism and subsequent replication [10,11]. *C*. *burnetii* actively controls infection by directing endosomes, vesicles, autophagosomes, and lysosomes to the PV, delivering nutrients, lipids, and proteins to the expanding vacuole that ultimately occupies most of the host cell cytosol [12,13]. Secretion of bacterial effector proteins into the host cytosol via a Dot/Icm type IV secretion system (T4SS) is essential for PV formation [14,15]. Some *C*. *burnetii* effectors contain sequences that resemble eukaryotic motifs and domains, bind host cell proteins, and manipulate host signaling to promote infection [16–18].

To support an intracellular lifestyle, *C*. *burnetii* manipulates several host cell signaling pathways. The pathogen activates Akt and Erk1/2 to promote host cell survival and allow completion of a lengthy infectious cycle [19]. *C*. *burnetii* also hijacks cyclic adenosine monophosphate (cAMP)-dependent protein kinase (PKA) signaling to support PV formation and prevent apoptotic cell death. When activated by cAMP, PKA binds and phosphorylates several downstream target proteins that modulate responses including cytokine production, apoptosis, and cytoskeletal remodeling. Differential phosphorylation of PKA substrates has been observed in *C*. *burnetii*-infected macrophages, and PKA signaling is indispensable for PV formation and intracellular bacterial replication [20]. Moreover, PKA phosphorylates Bcl-2-associated death promoter (Bad), resulting in sequestration of Bad to the PV and prevention of apoptosis [21]. Although most PKA substrates differentially phosphorylated during *C*. *burnetii* infection have not been characterized, they likely play unique roles in PV formation and Q fever pathogenesis.

PKA activity is critical for actin-related processes in eukaryotic cells and actin polymerization is involved in the intracellular lifestyle of multiple bacterial pathogens. A subset of intracellular bacteria recruit actin regulatory proteins to the bacterial cell surface to form actin tails that propel the pathogen through the cytosol and facilitate cell-to-cell movement without exposure to host immune cells. For example, *Listeria monocytogenes* produces ActA that recruits Arp2/3 and the PKA target protein vasodilator-stimulated phosphoprotein (VASP) to polymerize actin and form actin comets [22,23]. Manipulation of VASP by bacteria that do not use actin-based motility, such as *C*. *burnetii*, has not been reported. However, actin reorganization is essential for PV expansion in HeLa cells infected with avirulent *C*. *burnetii* [24]. In this study, we identified VASP as a PKA substrate that is preferentially activated during *C*. *burnetii* growth in human macrophages. Because PKA signaling is manipulated by *C*. *burnetii* [20,21] and VASP is a PKA substrate that regulates actin polymerization, we predicted that VASP is involved in PV formation and/or maintenance. Our findings indicate VASP is phosphorylated by PKA during infection in a T4SS-dependent fashion, and VASP activity is required for PV formation and *C*. *burnetii* replication in human macrophages. Additionally, we identified VASP residues (S157 and S239) required for PV formation and heterotypic fusion with other compartments. This study provides the first evidence of an intravacuolar bacterial pathogen usurping VASP function to promote replication vacuole formation.

## Results

### VASP is phosphorylated during *C*. *burnetii* growth in macrophages

**Fig 1:**
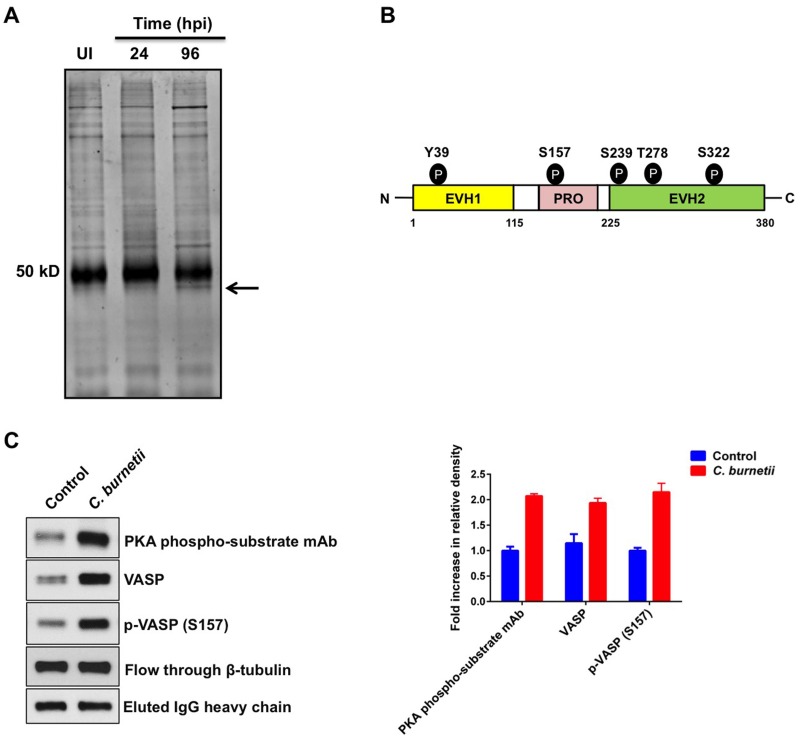
VASP is differentially phosphorylated during *C*. *burnetii* infection. THP-1 cells infected for 24 or 96 h and uninfected control cells were harvested and total protein immunoprecipitated with a PKA phospho substrate-specific antibody. (A) Immunoprecipitated proteins were subjected to Coomassie Blue staining and the arrow indicates a ~ 47 kDa protein only in infected cell samples. UI = Uninfected Cells. (B) Schematic of VASP showing five phosphorylation sites (Y39, S157, S239, T278, and S322) and EVH protein-protein interaction domains. PRO = proline rich region. (C) Immunoprecipitated proteins were subjected to immunoblot analysis using antibodies directed against VASP, phosphorylated VASP (S157), or PKA phospho substrates. Lysates were analyzed for β-tubulin to confirm equal protein loading. Agarose bead-bound PKA phospho substrate-specific antibody was eluted, immunoblotted, and probed with anti-IgG antibody to confirm equal amounts of antibody for immunoprecipitations. Densitometry analysis is shown in the graph and represents an average of three independent experiments. Error bars indicate the standard error of the mean. Control = uninfected cells. Increased levels of total and phosphorylated VASP (S157) are present in *C*. *burnetii*-infected cells.

We previously showed that PKA substrates are differentially phosphorylated during *C*. *burnetii* infection of human macrophages [20]. Moreover, PKA activation and phosphorylation of downstream targets occurs throughout intracellular growth (24–96 h post-infection (hpi)). To identify PKA target proteins with increased phosphorylation during infection, we immunoprecipitated PKA substrates from *C*. *burnetii*-infected THP-1 macrophage-like cells using a PKA phospho substrate-specific antibody. This antibody specifically recognizes proteins with phosphorylated Ser/Thr residues with arginine at the -3 and -2 positions (RRX**S**/**T**). At 24 and 96 hpi, immunoprecipitated proteins were analyzed by Coomassie blue staining. We consistently observed an increase in the levels of a ~47 kDa protein in *C*. *burnetii*-infected cells (Fig 1A). Mass spectrometry analysis identified this protein as eukaryotic vasodilator-stimulated phosphoprotein (VASP), a known PKA target [25]. VASP is involved in actin polymerization, contains distinct protein-protein interaction domains, and is regulated by phosphorylation at five residues (Fig 1B). Immunoblot analysis using a VASP-specific antibody confirmed increased VASP levels in proteins immunoprecipitated from infected cells (Fig 1C). PKA commonly phosphorylates VASP at S157 [26]. Therefore, we further assessed whether phosphorylated VASP (S157) was immunoprecipitated with the PKA phospho substrate-specific antibody. As shown in Fig 1C, immunoblot and densitometry analysis revealed an increase in phosphorylated VASP (S157) levels in immunoprecipitated samples collected from infected cells, indicating VASP is targeted by PKA during *C*. *burnetii* intracellular growth.

### Phosphorylated VASP levels increase coincident with PV expansion and *C*. *burnetii* replication

**Fig 2:**
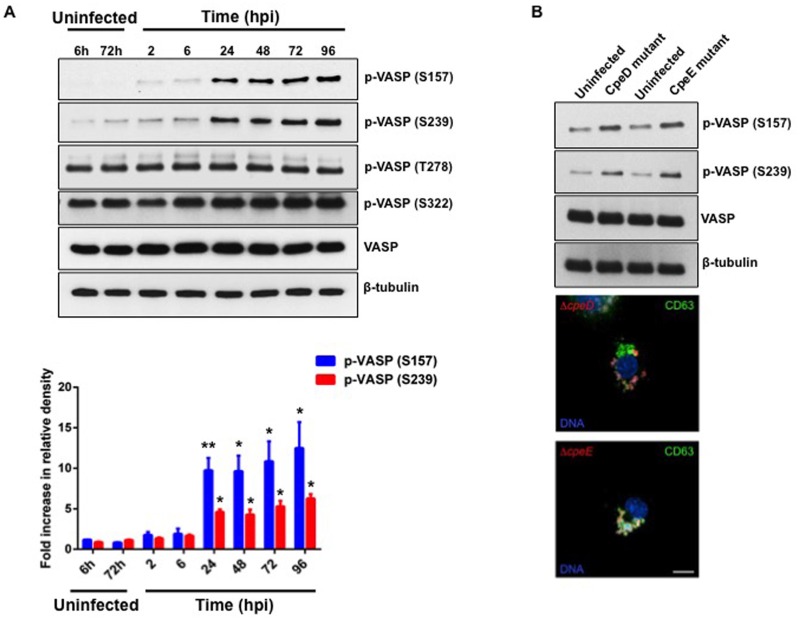
VASP phosphorylation levels increase during *C*. *burnetii* intracellular growth. (A) THP-1 cells were infected with *C*. *burnetii* for the indicated times and uninfected cells at 6 and 72 h were harvested as controls. (B) THP-1 cells were infected with *C*. *burnetii* deficient in CpeD or CpeE and harvested at 72 hpi for immunoblot or immunofluorescence analysis. Cell lysates were subjected to immunoblot analysis using antibodies directed against phosphorylated (S157, S239, S322, or T278) or total VASP. β-tubulin served as a loading control. Densitometric analysis is shown in the graph and represents an average of three independent experiments. Band intensities for all experimental conditions were normalized to β-tubulin levels, then compared to the average of uninfected controls. Error bars indicate the standard error of the mean. ** indicates p < 0.005 and * indicates p < 0.05 according to a Student’s *t* test. In panel B, cells were processed for microscopy using antibodies directed against *C*. *burnetii* (red) and CD63 (green). DAPI was used to stain DNA (blue). Levels of phosphorylated VASP (S157 and S239) increase significantly from 24–96 hpi by wild type and CpeD- (top) or CpeE-deficient (bottom) mutants that form a small, atypical PV, indicating that an expanded PV is not required for VASP phosphorylation.

VASP is involved in remodeling actin and is regulated by phosphorylation at Y39, S157, S239, S322, and T278. PKA, which plays a significant role in PV biogenesis [20], is known to phosphorylate VASP at S157, S239, and T278. Actin remodeling is a dynamic process involved in PV formation and/or maintenance [24]. Therefore, we predicted the kinetics of VASP phosphorylation may differ during early and late stages of infection. To test this hypothesis, we infected THP-1 cells with *C*. *burnetii* and collected cell lysates at 2–96 hpi. Immunoblot analysis using a specific antibody directed against phosphorylated VASP (S157) revealed low levels of phosphorylated protein in uninfected cells (Fig 2A). Cells infected with *C*. *burnetii* for 2 and 6 h also did not show a significant increase in levels of phosphorylated VASP, suggesting VASP activity is not required for early infection events, such as cell adherence, phagocytosis, and nascent phagosome formation. However, VASP phosphorylation levels increased significantly at 24 hpi and were maintained through 96 hpi (Fig 2A). Levels of total VASP remained unaltered in *C*. *burnetii*-infected cells, indicating VASP expression and turnover were not substantially altered by the pathogen. Furthermore, VASP can be phosphorylated at S239 by PKA or protein kinase G, and at T278 by adenosine monophosphate-activated protein kinase [25, 26]. Although *C*. *burnetii* infection did not trigger increased levels of phosphorylation at T278 or S322 in THP-1 cells, significantly increased levels of VASP phosphorylated at S239 were evident with similar timing to S157 phosphorylation (Fig 2A).

Although VASP phosphorylation increased prior to vacuole expansion (24 hpi), we sought to confirm that phosphorylation was not simply a consequence of the presence of a large vacuole. Cells were infected with *C*. *burnetii* deficient in CpeD or CpeE, which are both T4SS-secreted proteins. CpeD and CpeE mutant bacteria entered cells and were maintained in a vacuole much smaller than a typical PV (Fig 2B). However, increased VASP phosphorylation was apparent at 72 hpi with either CpeD- or CpeE-deficient *C*. *burnetii*, demonstrating that phosphorylation does not require a pre-formed large vacuole. Relative to uninfected cells, CpeD mutant-infected macrophages showed an ~ 1.9 fold increase in S157 and S239 phosphorylation. CpeE mutant-infected macrophages showed an ~ 8-fold and ~ 2.1-fold increase in S157 and S239 phosphorylation, respectively.

### PKA activity and a functional T4SS are required for increased VASP phosphorylation

**Fig 3:**
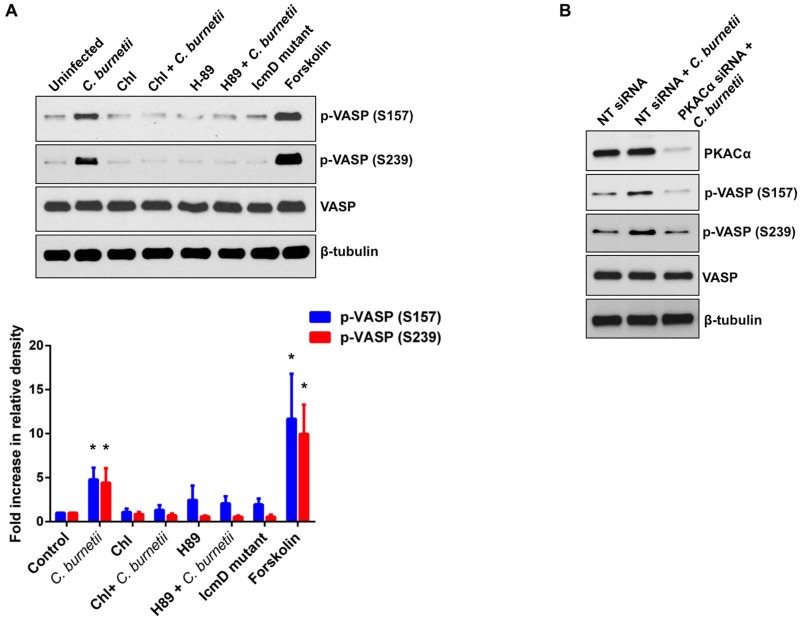
Increased VASP phosphorylation requires PKA and T4SS activity. (A) THP-1 cells were infected with wild type or IcmD mutant *C*. *burnetii* for 72 h. Indicated cells were treated with H89 to prevent PKA activity or chloramphenicol (Chl) to inhibit bacterial protein synthesis. Forskolin-treated cells served as a positive control for PKA activation. Cells were harvested at 72 hpi and subjected to immunoblot analysis using antibodies directed against phosphorylated (S157 and S239) or total VASP. β-tubulin served as a loading control. Densitometric analysis is shown in the graph and represents an average of three independent experiments. Band intensities for all conditions were normalized to β-tubulin levels, then compared to the average of uninfected controls. Error bars indicate the standard error of the mean. * indicates p < 0.05 according to a Student’s *t* test. VASP phosphorylation levels do not increase in the absence of PKA or T4SS activity, indicating this event is actively triggered by *C*. *burnetii* via the PKA cascade. (B) THP-1 cells were transfected with non-targeting (NT) or PKACα-specific siRNA, then infected with *C*. *burnetii* for 72 h, harvested, and immunoblotted with antibodies directed against PKACα, VASP, or phosphorylated VASP (S157 and S239). β-tubulin was used as a loading control. UI = Uninfected cells. Increased levels of phosphorylated VASP during infection are prevented in cells with decreased PKACα expression.

*C*. *burnetii* secretes effector molecules into the host cell cytosol using a Dot/Icm T4SS. PKA activation is triggered by secreted bacterial effectors, suggesting T4SS-defective *C*. *burnetii* should not trigger VASP phosphorylation. Therefore, we anticipated that inhibiting *C*. *burnetii* protein synthesis or antagonizing PKA signaling would prevent increased VASP phosphorylation. To test these predictions, we examined the status of VASP phosphorylation in the presence or absence of chloramphenicol or the PKA inhibitor H89 at 72 hpi, a time when robust VASP phosphorylation occurs. As shown in Fig 3A, treatment with H89 significantly reduced VASP phosphorylation, confirming VASP is a downstream target of PKA during infection. Treatment with the bacterial protein synthesis inhibitor chloramphenicol also abrogated increased VASP phosphorylation, supporting previous observations that induction of PKA signaling requires metabolically active *C*. *burnetii*. Similar to cells treated with H89 or chloramphenicol, IcmD mutant *C*. *burnetii*, which lacks a functional T4SS and cannot secrete effectors, did not alter VASP phosphorylation, indicating a secreted effector(s) promotes this signaling event (Fig 3A). As expected, treatment with the PKA activator forskolin triggered significant VASP phosphorylation at S157 and S239. The abundance of total VASP was not altered during infection or any inhibitor treatment. The requirement of PKA for VASP phosphorylation was further confirmed by silencing expression of the Cα subunit of PKA using a siRNA approach. This method depleted > 90% of PKACα relative to THP-1 cells transfected with non-targeting siRNA (Fig 3B). As expected, *C*. *burnetii* triggered increased VASP S157 and S239 phosphorylation in non-targeting siRNA-transfected cells. However, decreased expression of PKACα abrogated increased VASP phosphorylation without altering expression of total VASP. Relative to non-targeting siRNA-transfected cells, PKACα-silenced, *C*. *burnetii*-infected cells showed an ~ 66% and ~ 52% decrease in S157 phosphorylation and S239 phosphorylation, respectively. Together, these results indicate that *C*. *burnetii* triggers PKA- and T4SS-dependent phosphorylation of VASP at S157 and S239 in macrophage-like cells.

### Decreased VASP expression interferes with *C*. *burnetii* intracellular growth

**Fig 4:**
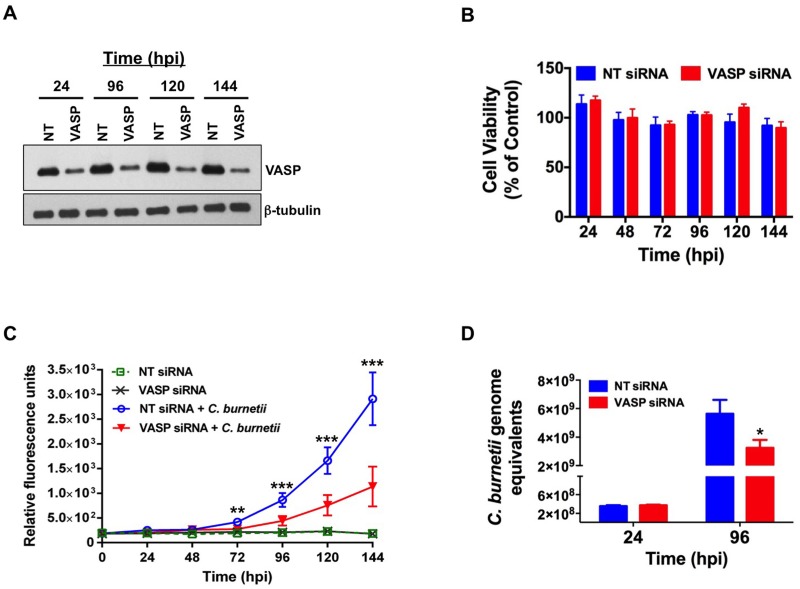
VASP activity is required for optimal *C*. *burnetii* intracellular replication. THP-1 cells were transfected with non-targeting (NT) or VASP-specific siRNA and harvested at 24–144 hpi. (A) Immunoblot analysis confirmed >80% knockdown of VASP production in VASP siRNA-transfected cells. (B) A viability assay demonstrated that no significant cell death occurred in VASP-depleted cells. (C) siRNA-transfected THP-1 cells were infected with *C*. *burnetii* expressing mCherry. Fluorescence was measured for 6 days after infection using a microplate reader (585/620 nm excitation/emission). (D) Bacterial genome equivalents were determined at 24 and 96 hpi using quantitative PCR. Each data point represents an average of seven replicates collected from two independent experiments, and error bars indicate standard deviation from the mean. * indicates p < 0.05, ** indicates p < 0.0005, and *** indicates p < 0.0001 according to a Student’s *t* test comparing NT and VASP-silenced cells infected with *C*. *burnetii*-mCherry. Depletion of VASP significantly impairs *C*. *burnetii* intracellular growth.

Based on the results above, we predicted that VASP is required for *C*. *burnetii* PV expansion and intracellular growth. To assess the importance of VASP function during infection, THP-1 cells were transfected with VASP-specific siRNA or non-targeting siRNA. Cell lysates were collected from 24–144 h post-transfection and analyzed by immunoblot to confirm VASP knockdown. VASP production decreased >80% using this approach (Fig 4A) and phosphorylated VASP (S157) was barely detectable at 24 and 96 h post-transfection. Importantly, VASP silencing did not alter viability of transfected cells at any time point tested (Fig 4B). siRNA-transfected cells were then infected with mCherry-expressing *C*. *burnetii* and bacterial growth was monitored by measuring fluorescence for six days. In VASP-depleted cells, a significant reduction in *C*. *burnetii* growth was observed relative to non-targeting siRNA-transfected cells (Fig 4C). We next confirmed that reduction of mCherry fluorescence correlated with genome equivalents representing bacterial numbers. THP-1 cells were infected with wild type *C*. *burnetii*, total DNA isolated at 24 or 96 hpi, and genome equivalents determined as previously described [27]. As shown in Fig 4D, no significant difference in bacterial numbers was observed at 24 hpi between control and VASP-depleted cells, indicating VASP is not required for bacterial uptake by THP-1 cells. However, the number of bacterial genomes at 96 hpi was ~ 40% lower in VASP-depleted cells, corresponding to mCherry fluorescence results and confirming VASP is necessary for optimal bacterial growth in macrophages.

**Fig 5:**
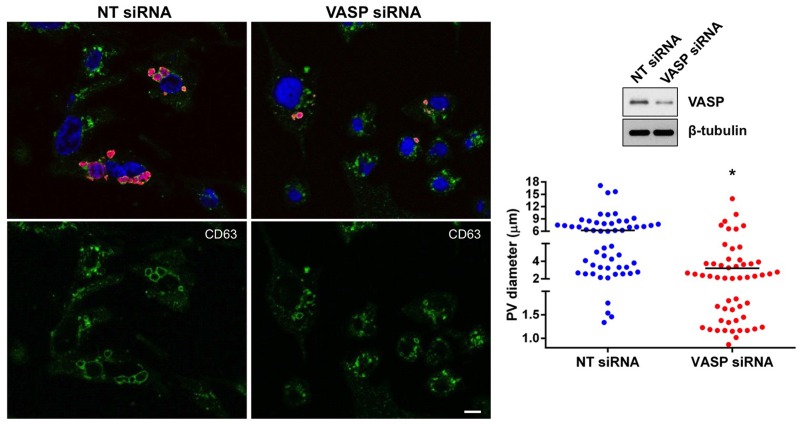
VASP activity is required for optimal PV formation. THP-1 cells were transfected with non-targeting (NT) or VASP-specific siRNA and knockdown confirmed by immunoblot. Cells were processed for confocal microscopy at 72 hpi. DNA was labeled with DAPI (blue), and CD63 (green) and *C*. *burnetii* (red) were detected with antibodies. Bar, 10 μm. The scatter plot displays PV diameter in NT or VASP siRNA-transfected cells. PV measurements were taken from at least 15 randomly selected fields. When multiple vacuoles were visible, the two largest vacuoles were measured in a single cell. The horizontal bar indicates average vacuole size. * indicates p < 0.0001 according to a Student’s *t* test comparing vacuole size in NT and VASP siRNA-transfected cells. VASP knockdown results in formation of smaller PV.

Next, we confirmed the growth curve analyses using confocal microscopy to monitor PV formation. The lysosomal marker CD63 was used to label the PV membrane. As shown in Fig 5, large (diameter > 6 μm), CD63-decorated PV were present in ~ 50% of non-targeting siRNA-transfected cells and only 7% of PV were smaller than 2 μm. In contrast, the number of vacuoles larger than 6 μm was reduced to 13% in VASP-silenced cells and 40% of PV were smaller than 2 μm (Fig 5). CD63 presence on small vacuoles in VASP-silenced cells indicated that VASP knockdown did not alter invasion events to prevent *C*. *burnetii* entry into the host cell or prevent phagolysosomal maturation. However, VASP silencing prevented vacuole expansion, potentially limiting available space and nutrients for replicating *C*. *burnetii*. Additionally, the impact of decreased VASP expression on PV formation was confirmed using a second set of VASP siRNA constructs. Together, these results indicate that VASP activity is required for optimal PV expansion and *C*. *burnetii* growth in human macrophages.

### Phosphorylated VASP levels increase during virulent *C*. *burnetii* growth in primary human alveolar macrophages

**Fig 6:**
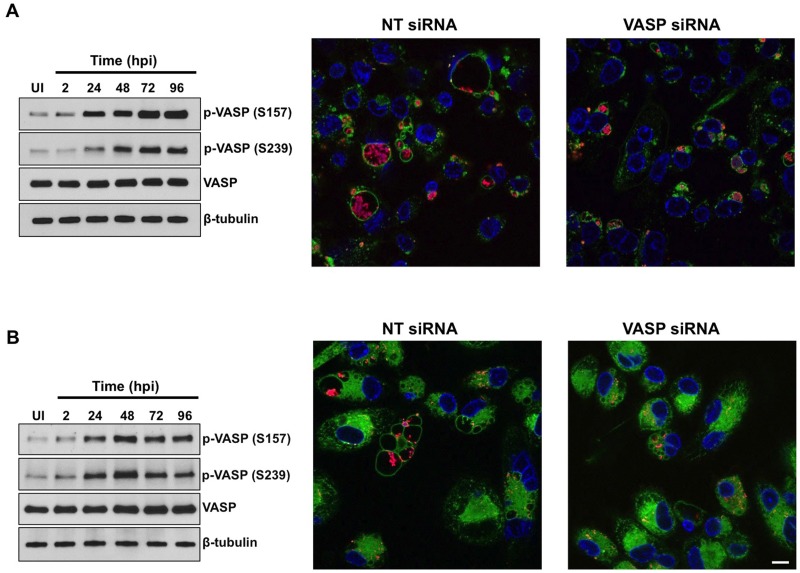
VASP activity is required for virulent *C*. *burnetii* PV formation. THP-1 cells (A) or primary hAMs (B) were infected with virulent *C*. *burnetii* and lysates collected at the indicated times. Immunoblotting was performed using antibodies directed against phosphorylated (S157 and S239) or total VASP (left panels). β-tubulin was used as a loading control. UI = Uninfected cells. Virulent *C*. *burnetii* triggered increased levels of phosphorylated VASP from 24–96 hpi in THP-1 cells and hAMs. In the middle and right panels, THP-1 cells (A) or hAMs (B) were transfected with non-targeting (NT) or VASP-specific siRNA. Cells were processed for confocal microscopy at 72 hpi. DNA was labeled with DAPI (blue), and CD63 (green) and *C*. *burnetii* (red) were detected with antibodies. Bar, 10 μm. VASP-depleted macrophages contain smaller PV than NT siRNA-transfected cells.

Avirulent and virulent *C*. *burnetii* produce different LPS structures, with virulent phase I LPS masking cell wall antigens that activate Toll-like receptors, preventing activation of the innate immune response [28]. Avirulent phase II *C*. *burnetii* is widely used for *in vitro* studies to characterize pathogen-host cell interactions [29]. However, results obtained from avirulent studies should be validated using virulent *C*. *burnetii*, as human disease is caused by phase I organisms. Similar to avirulent bacteria, THP-1 cells infected with virulent *C*. *burnetii* contained increased levels of phosphorylated VASP (S157 and S239) from 24–96 hpi without altering levels of total VASP (Fig 6A, left panel). siRNA-mediated knockdown of VASP expression substantially reduced PV expansion and resulted in smaller vacuoles (Fig 6A, middle and right panels). These results indicate that virulent *C*. *burnetii* uses host VASP for optimal PV formation. In addition to obtaining efficient silencing using individual siRNA, we observed similar PV formation results when cells were transfected with a mixture of VASP siRNA constructs. Although THP-1 macrophage-like cells are commonly used as an *in vitro* cellular model [17,30,31], *C*. *burnetii* preferentially infects and replicates within human alveolar macrophages (hAMs) during natural infection [32]. Additionally, alveolar macrophages express substantial levels of VASP [33]. Therefore, we isolated cells from human lungs post-mortem and infected primary hAMs with virulent *C*. *burnetii*. As shown in Fig 6B (left panel), infection triggered increased VASP phosphorylation (S157 and S239) from 24–96 hpi similar to THP-1 cells. Knockdown of VASP expression by siRNA interfered with PV expansion and cells contained smaller vacuoles relative to non-targeting siRNA-transfected cells (Fig 6B, middle and right panels). These results demonstrate the importance of VASP activity in a natural *C*. *burnetii* disease scenario.

### Mutation of VASP phosphorylated residues impairs PV formation

**Fig 7:**
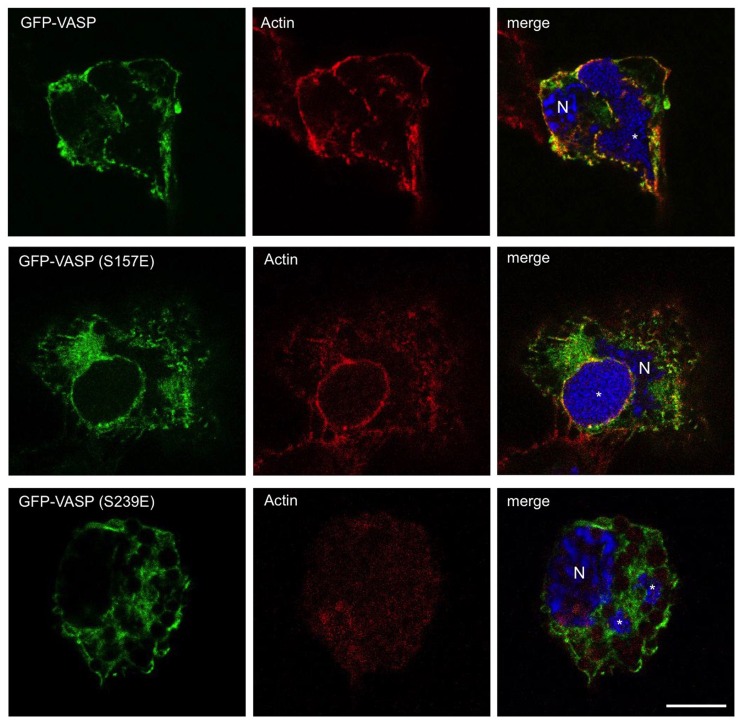
VASP S239E expression interferes with optimal PV formation. THP-1 cells were transfected with constructs encoding GFP-tagged wild type VASP (GFP-VASP), GFP-VASP (S157E), or GFP-VASP (S239E). At 72 hpi, cells were processed for fluorescence microscopy and DNA was stained with DAPI (blue). Actin was labeled with phalloidin (red). Bar, 10 μm. N = nucleus and * indicates the PV. GFP-VASP and GFP-VASP (S157E) localized around the PV with actin, while GFP-VASP (S239E) expression interfered with vacuole expansion and actin co-localization.

Phosphorylation of VASP at S157 and S239 regulates protein localization and actin polymerization, respectively [26]. We specifically tested whether motifs containing S157 or S239 are required for PV formation by expressing GFP-VASP constructs with individual residues mutated (S157E and S239E) in *C*. *burnetii*-infected THP-1 cells. A serine to glutamic acid mutation mimics the conformation of a phosphorylated serine with respect to negative charge, maintaining the structure of a phosphorylated protein [34]. However, the ability to bind target proteins that directly recognize phospho-serine motifs is prevented by this mutation. To examine functional effects of individual VASP mutants, we assessed VASP localization, actin arrangement, and PV formation in infected cells. As shown in Fig 7, large typical PV were present at 72 hpi in GFP-VASP and GFP-VASP (S157E)-expressing cells. Additionally, wild type and VASP (S157E) co-localized with actin around the PV. In contrast, expression of GFP-VASP (S239E) resulted in substantially smaller PV containing fewer *C*. *burnetii*. Furthermore, actin levels were reduced around the PV in GFP-VASP (S239E)-expressing cells. These results indicate proper S239 phosphorylation and regulation of distinct downstream target proteins is critical for infection-specific VASP functions potentially through interactions with a host or bacterial protein(s). Reduced actin polymerization may also destabilize the PV, resulting in smaller vacuoles.

**Fig 8:**
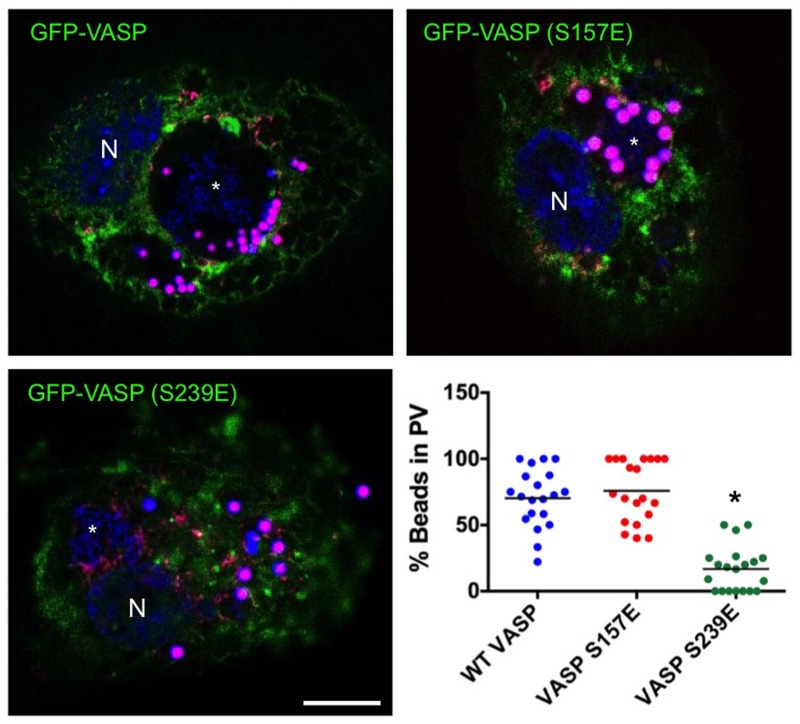
VASP (S239E) expression prevents phagosome trafficking to the PV. THP-1 cells were transfected with wild type (WT) GFP-VASP (GFP-VASP), GFP-VASP (S157E), or GFP-VASP (S239E), then infected with *C*. *burnetii*. Cells were incubated with fluorescent beads (violet) overnight and processed for confocal microscopy at 72 hpi. DNA was stained with DAPI (blue). Bar, 10 μm. N = nucleus and * indicates the PV in micrographs. The scatter plot displays the percentage of beads present within PV in GFP-VASP-, GFP-VASP (S157E)-, or GFP-VASP (S239E)-expressing cells. At least 20 cells from randomly selected fields were used for quantification. The horizontal bar indicates average percentage. * indicates p < 0.0001 according to a Student’s *t* test comparing wild type- and mutant VASP-expressing cells. Bead-containing phagosomes were directed to the PV in GFP-VASP- and GFP-VASP (S157E)-expressing cells. However, GFP-VASP (S239E) expression impaired bead trafficking to the PV.

PV generation involves numerous heterotypic fusion events with phagosomes, autophagosomes, and lysosomes [29]. To determine if VASP function is required for phagosome trafficking to the PV, THP-1 cells were infected with *C*. *burnetii* for 72 h, then incubated with fluorescent beads, a common technique for assessing PV heterotypic fusion with cellular compartments [35]. As shown in Fig 8, beads were delivered to the PV in cells expressing GFP-VASP or GFP-VASP (S157E). In contrast, beads were sequestered away from PV in cells expressing GFP-VASP (S239E), corresponding to the PV formation results observed above. These results indicate proper VASP S239 activity is critical for heterotypic fusion events during *C*. *burnetii* infection.

**Fig 9:**
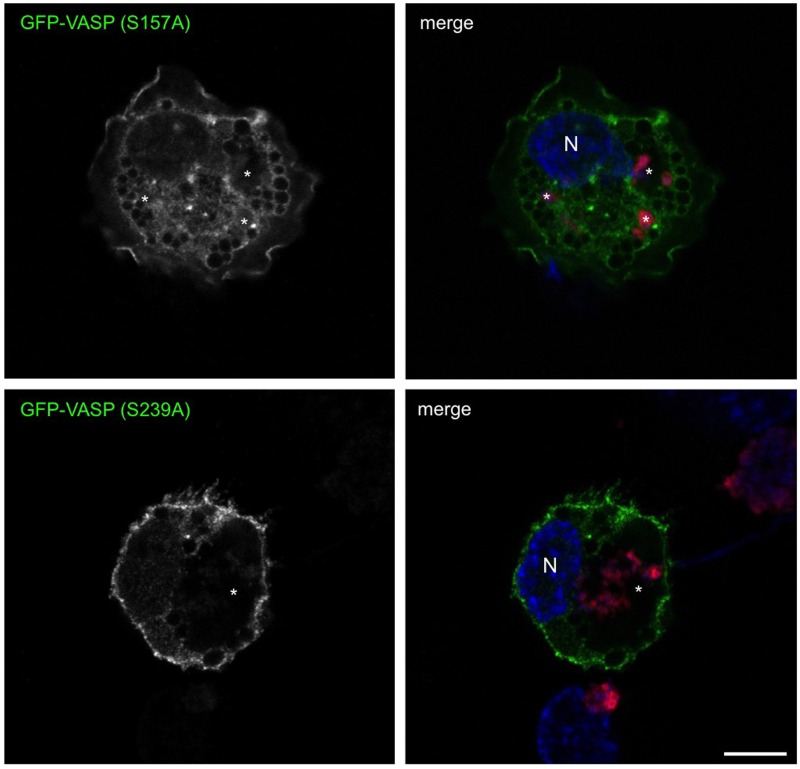
VASP (S157A) expression prevents typical PV formation. THP-1 cells were transfected with constructs encoding GFP-VASP (S157A) or GFP-VASP (S239A). At 72 hpi with *C. burnetii* (red), cells were processed for fluorescence microscopy and DNA was stained with DAPI (blue). Bar, 10 μm. N = nucleus and * indicates the PV. Results are representative of at least three separate experiments. GFP-VASP (S239A) expression allowed typical PV expansion, while GFP-VASP (S157A) expression interfered with vacuole expansion, indicating phosphorylation of S157 is critical for PV development.

To further assess the requirement of VASP phosphorylation for PV development, cells were transfected with constructs encoding phosphorylation-defective VASP mutants (S157A and S239A). In contrast to the phospho-mimetic mutants above, these proteins serve as dominant negative mutants and are not phosphorylated. As shown in Fig 9, cells expressing GFP-VASP or GFP-VASP (S239A) contained expanded PV while cells expressing GFP-VASP (S157A) harbored multiple small atypical PV. These results indicate that S157 phosphorylation is critical for PV expansion.

## Discussion

Here, we demonstrate, for the first time, that eukaryotic VASP can be exploited by an intracellular bacterial pathogen to promote replication vacuole formation. Successful PV formation by *C*. *burnetii* is necessary for development of Q fever and the pathogen actively manipulates host signaling using effector proteins secreted into the host cell cytosol via a Dot/Icm T4SS. Previous studies in our laboratory demonstrated that PKA is hijacked by *C*. *burnetii* to facilitate PV formation and prevent apoptotic cell death [20,21]. In this study, we identified VASP as a PKA substrate that is activated during infection by T4SS-proficient *C*. *burnetii*. VASP is an essential protein for actin remodeling; therefore, we hypothesize that VASP activity is required for actin-dependent PV expansion and/or maintenance in human macrophages.

As a member of the Ena/VASP protein family, VASP has an N-terminal EVH1 domain, C-terminal EVH2 domain, and proline-rich region (Fig 1B). The EVH1 domain binds to focal adhesion proteins that anchor VASP to the integrin complex of cell membranes [36,37]. The proline-rich region binds to SH3 and WW domain-containing proteins and profilin, which catalyze actin monomer formation and fast actin polymerization [38]. The EVH2 domain mediates F- and G-actin binding to regulate actin polymerization [39,40]. Anti-capping is one mechanism proposed for VASP-dependent actin polymerization where VASP binds to actin filament barbed ends and prevents recruitment of capping proteins, resulting in long actin filaments [41]. Additionally, VASP can inhibit Arp2/3-dependent actin filament branching; however, a molecular mechanism has not been characterized [23]. VASP dysfunction has been linked to many diseases including cancer, atherosclerosis and thrombosis [42].

VASP contains three major phosphorylation sites, S157, S239, and T278 that control numerous cellular functions. For example, phosphorylation by PKA at S157 facilitates localization to the cell periphery into focal adhesions [43], whereas phosphorylation at S239 and T278 regulates F-actin assembly [26]. During *C*. *burnetii* infection, prolonged VASP phosphorylation (24–96 hpi) occurs at S157 and S239. S157 is located adjacent to a proline-rich region and controls VASP translocation to the cell membrane, while exerting a minimal effect on F-actin polymerization [25,26,43–45]. S157 phosphorylation also blocks VASP interaction with Abl, SH3 domains, and Src proteins [26,46]. Phosphorylation of S239, which is located adjacent to a G-actin binding motif in the EVH2 domain, negatively regulates VASP anti-capping activity, resulting in shortened F-actin filaments, reduced F-actin accumulation, and filopodia formation [26,45,47,48]. Phospho-mimetic mutation of S239 reportedly decreases F-actin accumulation, does not efficiently co-localize with actin, and antagonizes adhesion and spreading of smooth muscle cells [49]. The timing of increased VASP phosphorylation during *C*. *burnetii* infection correlates with PV biogenesis and expansion, events that are regulated by T4SS effectors. Moreover, *C*. *burnetii* actively replicates during this time, and bacterial protein synthesis and T4SS activity are required to trigger increased VASP phosphorylation levels. Therefore, we predict a distinct secreted effector(s) hijacks PKA signaling to promote VASP phosphorylation.

PKA and protein kinase C (PKC) can phosphorylate VASP at S157 in human platelets [50] and protein kinase G preferentially phosphorylates the protein at S239 [45,50]. However, PKA phosphorylates S157 and S239 during *C*. *burnetii* infection as demonstrated by specific inhibitor and siRNA treatments. Four different PKA catalytic subunits, Cα, Cβ, Cγ, and X chromosome encoded protein kinase X (PRKX), have been identified in humans [51]. Using a confirmatory siRNA knockdown approach, it is clear that the PKACα subunit is required for S157 and S239 phosphorylation during infection. PKACα promotes breast cancer cell viability by inactivating the pro-apoptotic BCl-2-associated death promoter protein [52]. We previously showed that PKA promotes macrophage survival during *C*. *burnetii* infection, although the specific subunit responsible has not been defined. Considering an important role in cell survival, we anticipate PKACα is critical for PKA pro-survival signaling and VASP phosphorylation during infection.

A subset of intracellular pathogens, such as *Listeria monocytogenes*, recruit VASP to facilitate actin tail formation [53]. Resulting actin tails propel *L*. *monocytogenes* through the cytosol and to the cell surface, facilitating spread to other cells. Thus, hijacking VASP for actin-based motility is a critical part of the *L*. *monocytogenes* pathogenic life cycle. In contrast, some intracellular bacteria, such as *Shigella spp*., do not require VASP for actin-based mobility [54]. To our knowledge, the current study provides the first evidence of VASP manipulation by a non-motile, intravacuolar bacterium to control replication vacuole formation and promote intracellular replication. Indeed, siRNA-mediated VASP knockdown prevents typical *C*. *burnetii* growth and PV formation. Additionally, VASP activity is required during virulent *C*. *burnetii* infection of primary hAMs, suggesting the protein is necessary for optimal pathogen growth in the human lung. We predict that VASP-dependent actin rearrangements around the PV are required for vacuole stability and expansion. This prediction is supported by co-localization of GFP-VASP and actin at the PV membrane. Our macrophage infection results are consistent with reports suggesting actin is required for avirulent *C*. *burnetii* PV formation in HeLa cells [24]. Using F-actin depolymerizing chemical agents, Aguilera *et al*. showed that F-actin polymerization facilitates fusion between the PV and bead-containing phagosomes. Autophagosomes, lysosomes, and phagosomes continuously fuse with the PV, providing nutrients and membrane, and VASP may facilitate these actin-dependent vesicular fusion events. Additionally, actin may provide structural support for the maturing PV, allowing controlled expansion within the cytosol. A role for actin in structural support has been reported for *Chlamydia trachomatis* replication vacuole formation [55]. During growth in epithelial cells, actin cages form around the *C*. *trachomatis* inclusion, and disruption of actin polymerization diminishes vacuole membrane integrity. Therefore, it is possible that VASP is involved in growth of *C*. *trachomatis* and other intravacuolar pathogens by regulating formation of a nucleating complex at the replication vacuole membrane.

Phosphorylated VASP motifs recruit adaptor proteins that assemble protein complexes. For example, members of the 14-3-3 family, WD40 domain-containing F-box proteins, and WW domain-containing proteins form multimolecular signaling complexes through specific interactions with phospho-Ser/Thr motifs [56]. It is not known if similar proteins bind to VASP motifs to regulate macrophage responses to *C*. *burnetii*. However, ectopic expression of VASP (S239E) negatively impacts PV formation and reduces actin accumulation around the PV. In contrast, expression of VASP (S157E) does not alter PV formation or actin accumulation at the vacuole, consistent with reports that S157 does not significantly impact F-actin polymerization. During infection, VASP phosphorylated at S239 may localize to specific regions around the PV where controlled actin depolymerization could occur to provide space for vacuole expansion. Although VASP (S239E) has a negative charge, is functionally active, and negatively impacts actin polymerization, the mutant protein may not assemble phospho-Ser/Thr motif-binding protein complexes needed for PV formation. Moreover, VASP-mediated arrangement of actin ultrastructure around the PV may facilitate fusion of incoming vesicles and phagosomes with the PV. Indeed, bead trafficking results indicate S239 is critical for phagosome movement to the PV. These results do not prove that VASP plays a direct role in fusion events, but rather allows incoming phagosomes access to the PV along the cytoskeletal network.

Expression of VASP (S157A) severely impairs PV expansion, while VASP (S239A) expression allows normal vacuole development, demonstrating S157 phosphorylation is critical for infection. VASP phosphorylation at S157 prevents interactions with the SH3 domains of Abl, alpha II spectrin, and Src [46,57], and also regulates VASP cellular distribution. The dominant negative S157A mutant, which is not phosphorylated, may facilitate interaction with SH3 domain-containing proteins, preventing typical PV formation. The finding that expression of the phosphomimetic S157E mutant, which mimics phosphorylated VASP and provides conformational similarity, does not adversely impact PV formation supports the importance of S157 phosphorylation for optimal PV formation. Additionally, S157 phosphorylation minimally impacts actin filament formation [26]. Therefore, S157 phosphorylation may primarily act via regulation of other targets that contribute to optimal PV formation, and not by direct actin rearrangement. In contrast, expression of the dominant negative S239A mutant, has no significant impact on PV formation. Under physiological conditions, S239 is phosphorylated in small quantities and the level of phosphorylation is tightly regulated [26,47]. Therefore, PV-localized S239-phosphorylated VASP may reduce local F-actin levels, providing the necessary space for incoming cargo-laden vesicles and facilitating fusion events. It is also possible that infected macrophages regulate actin rearrangement via redundant mechanisms in the absence of S239-phosphorylated VASP.

In conclusion, *C*. *burnetii* hijacks host PKA signaling to phosphorylate VASP and facilitate PV formation. VASP phosphorylation requires bacterial protein synthesis and an active T4SS, indicating the pathogen actively targets this pathway. VASP may directly regulate actin polymerization dynamics, providing structural stability and physical space for the expanding PV needed for bacterial replication. Supporting this prediction, depletion of VASP negatively impacts intracellular bacterial growth and PV size. Phosphorylation at S157 and S239 is critical for VASP promotion of PV formation. Overall, this study provides the first evidence of a non-motile intravacuolar bacterial pathogen manipulating eukaryotic VASP to facilitate intracellular growth.

## Materials and Methods

### Bacteria and mammalian cell culture

Avirulent *C*. *burnetii* Nine Mile phase II (NMII; RSA439, clone 4), virulent Nine Mile phase I (NMI; RSA493), ΔCpeD, and ΔCpeE bacteria were cultured in acidified citrate cysteine medium (ACCM) at 37°C with 5% CO_2_ and 2.5% O_2_ for 7 days. Cultures were then washed and resuspended in sucrose phosphate buffer (pH 7.4). mCherry-expressing *C*. *burnetii* was grown in ACCM supplemented with chloramphenicol (3 μg/ml; Sigma). IcmD mutant *C*. *burnetii* (*icmD*::Tn) [14] was grown in ACCM supplemented with kanamycin (350 μg/ml). Construction of CpeD- and CpeE-deficient strains is described in S1 Text. All work with virulent *C*. *burnetii* was performed in the Centers for Disease Control and Prevention-approved biosafety level-3 facility at the University of Arkansas for Medical Sciences.

Human THP-1 monocytes (TIB-202; ATCC) were cultured in RPMI1640 medium (Invitrogen) supplemented with 10% fetal bovine serum (FBS; Invitrogen). Before infection, THP-1 cells were treated with phorbol 12-myristate 13-acetate (PMA; 200 nM) overnight for differentiation into macrophage-like cells. After replacing PMA-containing medium with fresh medium, THP-1 cells were infected with *C*. *burnetii* at a multiplicity of infection (MOI) of 10. To facilitate infection, plates were centrifuged at 900 rpm for 5 minutes, then incubated for 2 h at 37°C. Cells were washed with medium to remove excess *C*. *burnetii* and supplemented with fresh medium for the duration of the infection. Primary human alveolar macrophages (hAMs) were collected from lung tissue obtained post-mortem from the National Disease Research Interchange and maintained in DMEM/F12 containing 10% FBS as previously described [36]. Use of primary hAMs was assessed by the University of Arkansas for Medical Sciences institutional review board and deemed to not be human subjects research (#87788).

### Co-immunoprecipitation

THP-1 cells were infected with *C*. *burnetii* and harvested at 72 hpi in non-denaturing buffer (20 mM Tris-HCl pH 7.4, 0.1% Triton X-100, 150 mM NaCl, 2 mM NaF, 0.1% glycerol, and a protease and phosphatase inhibitor cocktail). Uninfected cells were processed as controls. Total protein was quantified using a DC protein assay (BioRad) and confirmed by immunoblot to detect β-tubulin. Immunoprecipitations (IPs) were performed using a Classical IP kit (Pierce). Lysates were pre-cleared by incubation with agarose resin for 1 h at 4°C. For immune complex preparation, 1 mg of total protein was incubated overnight with 10 μg of anti-PKA phospho substrate antibody at 4°C. Immune complexes were captured by incubation with protein A/G agarose beads for 1h at 4°C, then eluted in elution buffer (0.12 M Tris-HCl, 2% SDS, 20% glycerol, pH 6.8). Eluted PKA phospho substrate antibody was assessed by immunoblot and probing with anti-IgG to confirm equal amounts of capture antibody in control and infected samples.

### Immunoblot analysis

THP-1 cells in 6-well plates were harvested in lysis buffer (50 mM Tris, 1% sodium dodecyl sulfate (SDS), 5 mM EDTA, 5 mM EGTA, 1 mM sodium vanadate, protease and phosphatase inhibitor cocktails). Lysates were passed through a 26 gauge needle 10 times and quantified using the DC protein assay. Equal amounts of total protein were separated using 4–15% Mini-PROTEAN TGX gels (BioRad). Proteins were then transferred onto a polyvinylidene fluoride membrane (BioRad), and membranes blocked with 5% milk in Tris-buffered saline containing 0.1% Tween 20 (TBS-T). Membranes were probed with mouse anti-human VASP (BD Biosciences), rabbit anti-human p-VASP S157 (Cell Signaling), rabbit anti-human p-VASP S239 (Cell Signaling), rabbit anti-human p-VASP T278 (Sigma), rabbit anti-human p-VASP S322, or mouse anti-human β-tubulin (Sigma) primary antibodies diluted in TBS-T with 5% BSA. Anti-mouse or anti-rabbit IgG secondary antibodies conjugated to horseradish peroxidase (Cell Signaling) were used for chemiluminescence-based detection. Secondary antibodies were diluted in 5% milk-containing TBS-T and membranes incubated 1 h at room temperature. Reacting proteins were visualized using a WesternBright ECL kit (Advansta) and exposure to film.

### Modulation of PKA activity

Previously optimized protocols were used for chemical inhibitor treatments [20]. THP-1 cells in 6-well plates were infected with *C*. *burnetii* as described above. After 2 h, infectious inoculum was replaced with fresh medium and cells were treated with specific inhibitors or inducers. Media was replaced daily and chemicals were present throughout. Whole cell lysates were collected at the indicated times post-infection and processed for immunoblot analysis. H89 (10 μM; Sigma) was used to inhibit PKA activity and forskolin (10 μM; Sigma) was used to trigger PKA signaling. To inhibit intracellular *C*. *burnetii* protein synthesis, infected cells were treated with chloramphenicol (10 μg/ml).

### siRNA-mediated VASP knockdown and VASP mutant protein overexpression

Validated human VASP siRNA (5′- GGACCUACAGAGGGUGAAAdTdT -3') was used for VASP silencing [58], and non-targeting siRNA (5′-UGGUUUACAUGUCGACUAA-3') was used as a control for transfection experiments. THP-1 cells were nucleofected with VASP or non-targeting siRNA as previously described [59] with some modifications. THP-1 monocytes (3 X 10^6^) were resuspended in Human Monocyte Nucleofector Solution (Lonza). siRNA (1 μg) was mixed with cells, and cells were transfected using a Nucleofector 2b and program Y001 (Lonza). Following nucleofection, cells were transferred into fresh culture medium, then incubated 4 h at 37°C for recovery. Cells were treated overnight with PMA for differentiation into macrophage-like cells. After removing PMA-containing medium and replacing with fresh medium, monolayers were infected with *C*. *burnetii* as described above.

For siRNA-mediated knockdown of VASP expression in hAMs, DharmaFECT 1 transfection reagent (Thermo Scientific) was used according to the manufacturer’s instructions. hAMs were cultured on coverslips in 24 well plates. Transfection complex was formed using VASP siRNA (50 nM) and DharmaFECT 1 reagent, then added to cells drop wise and incubated overnight for siRNA uptake. Cells were washed once, supplemented with fresh medium, and infected with *C*. *burnetii* as indicated above.

For ectopic expression, full length human VASP, S157A, S239A, or S157E mutant cDNA cloned into pEGFP-N1, which were previously characterized for expression of GFP-VASP and GFP-VASP (S157E) [60], were used. Expression of GFP-VASP and GFP-VASP (S239E) was achieved using full length VASP or S239E mutant cDNA cloned into pCMV6-AC-GFP (OriGene). THP-1 cells were nucleofected with 1 μg of each plasmid as described above.

### Cell viability analysis

Viability was determined using a Cell Counting Kit-8 (Dojindo Laboratories) according to the manufacturer’s instructions. siRNA-transfected cells were left uninfected or infected for the indicated times in 96-well plates. At each time point, WST-8 (2-(2-methoxy-4-nitrophenyl)-3-(4-nitrophenyl)-5-(2,4-disulfophenyl)-2H-tetrazolium, monosodium salt) reagent was added to wells and incubation continued for 4 h at 37°C. Following measurement of the A_450_ of cultures, viability was calculated using the following formula: {(A_test_-A_background_)/(A_control_-A_background_) x 100}, where A = absorbance at A_450_, background = media alone, and control = non-transfected cells.

### Confocal fluorescence microscopy

THP-1 cells were plated on 12 mm diameter circular cover glasses (Fisher) in 24-well plates. After treatments and infections, cells were fixed and permeabilized with 100% ice-cold methanol for 3 min and blocked with 0.5% bovine serum albumin (BSA) in PBS (pH 7.4) for 1 h at room temperature. For actin detection, cells were fixed with 4% formaldehyde for 15 min and blocked with 0.5% BSA containing 0.3% Triton X-100 for 1 h. Cells were incubated with mouse anti-CD63 (BD Biosciences) and rabbit anti-*C*. *burnetii* primary antibodies in 0.5% BSA for 1 h at room temperature. After washing with PBS, cells were incubated with Alexa Fluor 488-conjugated anti-mouse IgG and Alexa Fluor 594-conjugated anti-rabbit secondary antibodies (Invitrogen). Indicated samples were treated with Alexa Fluor 594-labeled phalloidin (Invitrogen) for 30 min at room temperature to detect actin. In phalloidin-treated samples, CD63 primary antibody was detected with Alexa Fluor 633-conjugated anti-rabbit secondary antibody. Cells were incubated with DAPI for 5 min at room temperature and mounted with MOWIOL (Sigma). Confocal imaging was performed with a Nikon C2si microscope and data were analyzed using NIS-Elements software (Nikon). PV diameter measurements were taken from at least 15 randomly selected fields. When multiple vacuoles were visible, the two largest vacuoles were measured in a single cell and average vacuole size calculated for all fields.

### Fluorescent bead trafficking assay

THP-1 cells were transfected with constructs encoding GFP-VASP or serine mutants (S157E or S239E), then infected with *C*. *burnetii* as described above. 0.5 million fluorescent 1.0 μm polystyrene microsphere beads (Invitrogen) were added to cells on coverslips and incubated overnight at 37°C. Cells were washed with media to remove excess beads. At 72 hpi, cells were fixed with 4% paraformaldehyde and processed for confocal microscopy as described above.

### Growth curve analysis

For fluorescence-based growth curves, THP-1 cells were transfected with non-targeting or VASP siRNA as described above. Cells were then cultured in glass flat bottom 96 well black plates. After PMA treatment and removal, cells were infected with mCherry-expressing *C*. *burnetii* overnight (MOI = 10). Medium was then replaced with fresh medium and intracellular *C*. *burnetii* growth monitored by fluorescence intensity using a BioTek Synergy H1 microplate reader (585 nm excitation/620 nm emission) for 6 days post-infection.

For determination of genome equivalents, infected THP-1 cells were harvested in media by centrifugation (10,000 x g). Cells were disrupted by vortexing with microbeads and total DNA was extracted using an UltraClean Microbial DNA kit (MoBio Laboratories). 10 ng of total DNA was used for quantitative PCR. Previously optimized primers [27] designed to amplify *C*. *burnetii dotA* were used with a Power SYBR Green PCR master mix (Applied Biosystems). pCR2.1-*dotA* was serially diluted and used to generate a standard curve and calculate *C*. *burnetii* genome copies.

### Densitometry and statistical analysis

Immunoblots were scanned in gray scale with a resolution of 300 dpi and band intensities quantified using ImageJ software (version 1.48v). Band intensities were normalized to β-tubulin levels. All experiments were performed at least in triplicate. Statistical significance between experimental and control groups was calculated using a Students *t* test and Prism 6 software (GraphPad). Results were considered statistically significant when p < 0.05.

## Supporting Information

S1 Text Construction of Δ*cpeD* and Δ*cpeE* mutants. (DOCX)
