# Supplementary material for: Vasodilator-Stimulated Phosphoprotein Activity Is Required for Coxiella burnetii Growth in Human Macrophages
Source: PLoS Pathog. 2016 Oct 6;12(10):e1005915. doi: 10.1371/journal.ppat.1005915 (PMC5053435; doi:10.1371/journal.ppat.1005915)
Supplement: S1 Text — (DOCX) [file ppat.1005915.s001.docx]

**Supplemental Methods**

**Construction of Δ*cpeD* and Δ*cpeE* mutants**

The 5′ and 3′ flanking regions of *cpeD* and *cpeE* were amplified from Nine Mile RSA439 genomic DNA by PCR using the oligonucleotides pairs CpeD-5′F/CpeD-5′R and CpeD-3′F/CpeD-3′R or CpeE-5′F/CpeE-5′R and CpeE-3′F/CpeE-3′R, respectively. The corresponding 5′ and 3′ fragments were cloned into BamHI/SalI-digested pJC-CAT using In-Fusion (BD ClonTech), resulting in the formation of an internal NdeI or AgeI site between the 5′ and 3′ regions and the creation of pJC-CAT::CpeD-5′3′ and pJC-CAT::CpeE-5′3′, respectively. The *1169^P^*-Kan cassette was amplified from pJB-Kan by PCR with P1169-Kan-NdeI-KO-rev-F and P1169-Kan-NdeI-KO-rev-R or P1169-Kan-AgeI-KO-rev-F and P1169-Kan-AgeI-KO-rev-R oligonucleotides and cloned into NdeI or AgeI digested pJC-CAT::CpeD-5′3′ or pJC-CAT::CpeE-5′3′ to create pJC-CAT::CpeD-5′3′-Kan and pJC-CAT::CpeE-5′3′-Kan, respectively.

Targeted gene deletions were created in *cpeD* and *cpeE* as previously described [1]. Briefly, *C. burnetii* RSA439 was transformed with ~16 μg of pJC-CAT::CpeD-5′3′-Kan or pJC-CAT::CpeE-5′3′-Kan and primary integrants obtained following selection with chloramphenicol and kanamycin. Primary integrants were then treated with 1% sucrose in the presence of kanamycin to force removal of *sacB*-encoding plasmid co-integrants containing *cpeD* or *cpeE*, resulting in the kanamycin-marked deletion strains, Δ*cpeD* and Δ*cpeE* respectively. Clonal deletion mutants were then obtained via plating on ACCM2-agarose as previously described [2].

**Primers:**

CpeD-5'F - CGGTACCCGG**GGATCC**CACGAATGTACGATGGGATG

CpeD-5'R - CACC**CATATG**CGACGCGAGCGCATCGCTCAAAGCCTTACTTAATTTACC

CpeD-3'F - CGTCG**CATATG**GGTGCGCATGCTTAACGTGAAAGAAGGATTTTTG

CpeD-3'R - GAACCTGTTT**GTCGAC**CGGGATTTCTTTTCAAGCTC

CpeE-5'F - CGGTACCCGG**GGATCC**AACGGGTATATGACTTTAGGACAAG

CpeE-5'R - GCACC**ACCGGT**CGACGTCGCCCAAAAATCCTTCTTTCACGTTAAG

CpeE-3'F - CGTCG**ACCGGT**GGTGCCGAGGCAATCGCAAAATTTATCGGATATACC

CpeE-3'R - GAACCTGTTT**GTCGAC**GTGAAGTATTACGCATATTTATCTCCG

P1169-Kan-NdeI-KO-rev-F - CGCTCGCGTCG**CATATG**ATGGCTTCGTTTCGCAGCG

P1169-Kan-NdeI-KO-rev-R - GCATGCGCACC**CATATG**TTATCAGAAGAACTCGTCAAGAAGG

P1169-Kan-AgeI-KO-rev-F - GGCGACGTCG**ACCGGT**ATGGCTTCGTTTCGCAGCGAACTTGG

P1169-Kan-AgeI-KO-rev-R - CCTCGGCACC**ACCGGT**TTATCAGAAGAACTCGTCAAGAAGGC

**References**

1. Beare PA, Larson CL, Gilk SD, Heinzen RA (2012) Two systems for targeted gene deletion in *Coxiella burnetii*. Appl Environ Microbiol 78: 4580-4589.

2. Omsland A, Beare PA, Hill J, Cockrell DC, Howe D, et al. (2011) Isolation from animal tissue and genetic transformation of *Coxiella burnetii* are facilitated by an improved axenic growth medium. Appl Environ Microbiol 77: 3720-3725.
